# Interpersonal Sensitivity as Mediator of the Relations Between War Experiences and Mental Illness in War-Affected Youth in Northern Uganda: Findings From the WAYS Study

**DOI:** 10.1037/trm0000145

**Published:** 2018-04-26

**Authors:** Kennedy Amone-P’Olak, Ask Elklit

**Affiliations:** 1 Department of Psychology, University of Botswana, and National Center for Psychotraumatology, University of Southern Denmark; 2 National Center for Psychotraumatology, University of Southern Denmark

**Keywords:** interpersonal sensitivity, PTSD, depression, psychotic symptoms, war-affected youth

## Abstract

The pathways from war experiences to mental health problems are poorly understood. The current study aims to assess the role of interpersonal sensitivity in the relations between war experiences and mental health problems based on data from the *W*ar-*A*ffected *Y*outh *S*urvey cohort study. The *W*ar-*A*ffected *Y*outh *S*urvey is an ongoing research project of formerly abducted children in Northern Uganda assessing their war experiences and the risk and protective factors in the development of mental health problems. Mediation of the relations between war experiences and mental health problems by interpersonal sensitivity was analyzed using structural equation modeling. War experiences were related to posttraumatic stress disorder through interpersonal sensitivity accounting for 55% of the variance in their relations, to depression/anxiety through interpersonal sensitivity accounting for 89% of the variance in their relations (i.e., near complete mediation), and to psychotic symptoms through interpersonal sensitivity accounting for 53% of the variance in their relations. The direct relation between war experiences, on the one hand, and posttraumatic stress disorder and psychotic symptoms, on the other hand, attenuated but remained statistically significant. For depression/anxiety, the direct relationship ceased to be significant after including interpersonal sensitivity in the model. Interpersonal sensitivity is an important determinant of long-term mental health problems in war-affected youth. Interventions to improve mental health should target youth with high scores on interpersonal sensitivity. Cognitive–behavioral therapy to recognize and change cognitive schemas in youth prone to interpersonal sensitivity is recommended.

Numerous studies have consistently reported a high prevalence of mental health problems in the aftermath of war (Karam & Ghosn, 2003; Machel, 2001; Priebe et al., 2010; Steel et al., 2009). In a systematic review of studies conducted with refugees and other war-affected populations, Steel and colleagues (2009) found that about 30% of the survivors met the criteria for posttraumatic stress disorder (PTSD) and depression (Steel et al., 2009). Although the experience of war is linked to mental disorders, survivors who meet the criteria for disorders are relatively few, with substantial differences seen in how well individuals adapt to postwar environments.

A number of factors have been proposed to explain the variations in postwar mental health outcomes and adaptation. These factors include the type of war events experienced (Amone-P’Olak, Ovuga, Croudace, Jones, & Abbott, 2014; Johnson & Thompson, 2008; Kohrt et al., 2008) and coping styles (Amone-P’Olak, Garnefski, & Kraaij, 2007). Similarly, the aftermath of war is suggested to be rife with environmental stressors that are suggested to explain continued mental ill-health in war-affected populations (Amone-P’Olak, Otim, Opio, Ovuga, & Meiser-Stedman, 2014; Amone-P’Olak, et al., 2014; Fernando, Miller, & Berger, 2010; Kohrt et al., 2008; Miller & Rasmussen, 2010). Besides the type of war experiences, postwar environmental stressors have been linked to adverse social consequences such as poor emotional regulation (Amone-P’Olak et al., 2007), impaired ability to relate to others in the community, diminished trust, and interpersonal sensitivity (Amone-P’Olak, 2008; Cloitre, Stovall-McClough, Zorbas, & Charuvastra, 2008). Yet, hardly any studies have been conducted to assess the social consequences of war, such as increased sensitivity to developing a mental disorder among survivors of war, especially in Africa. Exploration of the causal pathways through which war experiences impact on survivor’s mental health can inform strategies to mitigate adverse outcomes from a public health point of view.

It is possible that the pathway through which war experiences may influence psychosocial outcomes is interpersonal sensitivity. The concept of interpersonal sensitivity was first developed by Boyce and Parker (1989), who defined it as “undue and excessive awareness of, and sensitivity to, the behavior and feelings of others” (p. 342). In the context of the war-affected youth, some of whom perpetrated horrendous atrocities against the civilian population to which they have been reintegrated, it has been defined as sensitivity to perceived or actual behaviors and feelings of others toward them that may define their mental health outcomes. Subsequent studies have reported that interpersonal sensitivity is linked to exposure to traumatic experiences, including war trauma (Hauff & Vaglum, 1994; Nickerson, Priebe, Bryant, & Morina, 2014), with exposure to war often associated with higher levels of interpersonal sensitivity (Hagenaars, Fisch, & van Minnen, 2011; Nickerson et al., 2014). Furthermore, interpersonal sensitivity has been linked to negative mental health outcomes, including PTSD, depression, and anxiety (Hauff & Vaglum, 1994; Nickerson et al., 2014). Responses such as lack of trust and interpersonal insensitivity may be adaptive, albeit in the short run, in the aftermath of war, especially where stigma and poor community relations trigger traumatic reminders and threats to war survivors (Nickerson et al., 2014). Consequently, it is possible that interpersonal sensitivity might explain continued experience of mental health problems such as PTSD, depression, and psychosis.

For 2 decades (1986–2006), Northern Uganda experienced a brutal war in which about 50,000 people were abducted, including an estimated 30,000 children, and about two million people internally displaced (UNICEF, 1998). Of the 30,000 children abducted, about 30% were young girls, 65% of them reported sexual abuse, and about 30% of the girls returned from rebel captivity with children fathered by rebel soldiers and commanders (Amone-P’Olak, 2005; Amone-P’Olak et al., 2016; Amone-P’Olak, Ovuga, & Jones, 2015). In rebel captivity, the abductees witnessed violence and were tortured, injured, involved in combat, used as human shields, and forced to mutilate, injure, or kill fellow abductees or civilians (Amone-P’Olak, 2004, 2009; Derluyn, Broekaert, Schuyten, & De Temmerman, 2004). All of these war experiences have been associated with mental health problems such as PTSD, psychotic symptoms, depression, anxiety, somatic complaints, and conduct problems (Amone-P’Olak, Jones, Abbott, et al., 2013; Betancourt et al., 2010; Boothby, 1996; de Jong, 2002; Derluyn et al., 2004; Dokkedahl, Oboke, Ovuga, & Elklit, 2015).

Furthermore, the war in Northern Uganda was fought in the community and sometimes victims knew the perpetrators. Consequently, postwar environment is fraught with personal vendetta, lack of trust, and interpersonal sensitivity, perceived or real (Nickerson et al., 2014). Although postwar interpersonal sensitivity may be unfounded, perceptions of it may lead to mental health problems, as demonstrated by previous findings (Hauff & Vaglum, 1994; Vidyanidhi & Sudhir, 2009; Wilhelm, Boyce, & Brownhill, 2004). Moreover, interpersonal sensitivity has the potential to disrupt social relations and daily functioning, as an outburst of anger and preoccupation with previous traumatic experiences may, in turn, engender further anger-related reactions and personal threats, leading to poor social relations and mental illness (Nickerson et al., 2014). Interpersonal sensitivity is suggested to be common, especially in postconflict environments, where obsession with previous political, social, and economic prejudices, as well as preoccupation with revenge, may be rampant (Lopes Cardozo, Vergara, Agani, & Gotway, 2000). Previous studies on the war in Northern Uganda reported a high prevalence of anger-related reactions and feelings of revenge (Murphy, Elklit, Dokkedahl, & Shevlin, 2016, 2017). Similar studies with war-affected populations and refugees have also suggested that the postwar environment is fraught with anger-related reactions (Brooks, Silove, Steel, Steel, & Rees, 2011; Hamama-Raz, Solomon, Cohen, & Laufer, 2008; Hinton, Hsia, Um, & Otto, 2003; Silove et al., 2009), which are linked to human rights violations and poverty (Brooks et al., 2011; Silove et al., 2009). Considering the huge costs to the criminal justice system and health care, the insecurity associated with anger and violence, and the associated link to mental health problems, empirical research that illuminates the path from war experiences to mental illness is imperative.

Although previous studies have demonstrated the adverse effects of war experiences on mental health and mental health problems with interpersonal sensitivity, it is possible that interpersonal sensitivity may explain the continued mental health problems among war-affected youth. Consequently, this study aims to assess the extent to which interpersonal sensitivity explains the associations between war experiences and mental health outcomes (PTSD, depression/anxiety, and psychotic symptoms) in war-affected populations. Data in this study were drawn from an ongoing *W*ar-*A*ffected *Y*outh *S*urvey (WAYS) in Northern Uganda. The WAYS study aimed to assess the long-term risk and protective individual, family, and community factors involved in the development and sustenance of mental health problems in war-affected youth in Northern Uganda. Based on the literature reviewed earlier, it is hypothesized that interpersonal sensitivity would mediate the associations between war experiences and mental health outcomes in formerly abducted youth in Northern Uganda.

## Method

### Design and Sample

The WAYS study used a longitudinal cohort design and recruited war-affected youth from five districts in Northern Uganda (Gulu, Amuru, Nwoya, Pader, and Kitgum) severely affected by the Lord’s Resistance Army (LRA) war. The youths were organized into several groups to promote social support and facilitate access to programs designed for war-affected populations. Previously, the United Nations Children’s Fund (UNICEF) compiled a list of children who had been abducted and forced into military service by LRA rebels from all districts of Northern Uganda. Other nongovernmental organizations and government departments used the list to distribute relief items to enable the formerly abducted youth to resettle in their respective communities. Based on the UNICEF list, the youth who met the following inclusion criteria were included in the WAYS study: (a) history of abduction by rebels, (b) having lived in rebel captivity for at least 6 months, and (c) were now aged between 18 and 25 years. Altogether, 650 participants who met the inclusion criteria were asked through their local leaders to participate. In the end, data were gathered from 539 youth at baseline, representing 83% of those eligible for the study. Details of participants in the WAYS study cohort can be found elsewhere (Amone-P’Olak, Jones, Abbott, et al., 2013). Baseline assessment was conducted from June to September 2011, 6 years after the conflict ended.

### Data Collection

Research assistants collecting data for the WAYS study were all university graduates with extensive training in data collection and interviewing skills. The research assistants were provided with training on the background of the WAYS study and further trained on how to conduct interviews. The research assistants were fluent in speaking and writing the native language of the participants (Luo) and the English language. Data collection took place in participants’ homes, nearby trading centers, or community halls. The data collected were on demographic characteristics; participants’ experiences before, during, and after the war; individual factors (e.g., interpersonal sensitivity); and mental health outcomes (PTSD, symptoms of depression/anxiety, and psychotic symptoms). The questionnaire took 30–45 min to complete.

The research assistants were accompanied by a clinical psychiatric officer who could take care of any mental health emergencies where there was a possibility for harm and who could make referrals to the regional referral hospital. Informed consent was obtained from all participants in accordance with ethical guidelines and approval from Gulu University Institutional Review Board and Uganda National Council for Science and Technology.

### Measures

The assessment of psychological outcomes using measures developed in Western societies is often difficult due to cultural differences and a lack of standardized measures in nonwestern societies that carry similar meaning (Bracken, Giller, & Summerfield, 1995). Accordingly, both standardized and locally developed measures were used in the current study.

#### War experiences

In this study, the UNICEF B&H (Bosnia and Herzegovina) Postwar Screening Survey (UNICEF, 2010) was used to assess exposures to different war events. The instrument was modified to reflect the local context of the conflict in Northern Uganda. For instance, questions on knowledge of, witnessing, and being sexually assaulted and/or abused were added. Finally, the modified instrument comprised 52 items on various types of war experiences. These war experiences included personal harm (six items, e.g., physical torture), witnessing general war violence (11 items, e.g., witnessing killing), sexual abuse (one item), and involvement in hostilities (two items, e.g., fighting with an army or warring faction). Other exposures included separation (two items), deaths (seven items, e.g., deaths of parents, siblings, or extended family members), material loss (four items), physical threat to self (five items), harm to loved ones (four items), physical threat to relatives or loved ones (four items), displacement (five items), and drug and substance abuse (one item). The war experiences were dichotomously coded for occurrence (1) versus absence (0). The war experiences were assessed at baseline.

#### Impact of Events Scale

The Revised Impact of Events Scale (IES-R) was used to assess symptoms of PTSD at follow-up (Horowitz, Wilner, & Alvarez, 1979; Weiss & Marmar, 1997). These symptoms were anchored to war-related traumatic experiences. The IES-R is a 22-item scale that indicates severity of PTSD symptoms with a Likert response format ranging from 0 (*not at all*) to 4 (*extremely*). Total symptom scores for each of the symptom’s clusters (Reexperiencing, Avoidance, and Hyperarousal) were computed by adding up the relevant item scores. The authors reported high test–retest reliabilities and internal consistencies of the three subscales, with alpha coefficients ranging from 0.79 to 0.92 (Horowitz et al., 1979; Weiss & Marmar, 1997). In the current study, the IES-R demonstrated high internal consistency values for the total scale as well as the three subscales, ranging from α = .81 to α = .89.

#### Mental health outcomes

Subscales from the Acholi Psychosocial Assessment Instrument (APAI), which is a modified version of the African Youth Psychosocial Assessment Instrument, were used in the current study. The subscales included Depression/Anxiety Symptoms (18 items), Somatic Complaints (three items), and Conduct Problems (10 items). APAI is a field-based measure previously developed for use in Northern Uganda (Betancourt et al., 2009). In APAI, depression and anxiety were a mixed set of items appearing as one scale. The Depression/Anxiety scale was represented by questions that indicate behavior specific to depression/anxiety, such as *I have lots of worries*, *I sit alone*, *I think about suicide*, and so forth. In this study, the Cronbach’s α values were 0.89 for the combined depression/anxiety items. Depression and anxiety symptoms commonly co-occur. Consequently, the questionnaire items that assessed depression and anxiety psychopathology were mixed together in one scale for common mental health problems, thus preventing them from being considered separately as distinct outcomes. Previous studies also showed a strong overlap among items in the Depression and Anxiety subscales (Brodbeck, Abbott, Goodyer, & Croudace, 2011). For each question, responses were scored from 0 to 3, where 0 = *never*, 1 = *rarely*, 2 = *sometimes*, and 3 = *always*. The mental health problems were assessed at follow-up.

#### Psychotic symptoms

Four items indicative of psychotic symptoms (i.e., hallucinations, delusions, and persecutory feelings) were used in the current study: (a) sometimes I hear voices or see things other people do not see, (b) sometimes I feel that I have special powers, (c) sometimes I think that people are listening to my thoughts or watching me when I am alone, and (d) sometimes I think that people are against me. Hallucinations, delusions, and persecutory feelings are all common characteristics of psychotic symptoms. The items were scored from 0 to 3, where 0 = *never*, 1 = *rarely*, 2 = *sometimes*, and 3 = *always*. The psychotic symptoms scale had good psychometric properties (Cronbach’s α = .71). Psychotic symptoms were assessed at follow-up.

#### Brief Symptom Inventory

The Brief Symptom Inventory measures different domains of psychological distress experienced in the previous week (Derogatis & Melisaratos, 1983). The Brief Symptom Inventory scale consists of 53 items scored on a 5-point response format ranging from 0 (*not at all*) to 4 (*extremely*). The interpersonal sensitivity subscale is indicated by four items (e.g., “Feeling that people are unfriendly or dislike you”). In this study, only the Interpersonal Sensitivity subscale is used in the analysis and was assessed at follow-up. The internal consistency of the Interpersonal Sensitivity subscale in the current study was acceptable at α = 0.82.

### Statistical Analyses

First, correlations among demographic, predictor, and outcome variables were computed. Second, structural equation modeling (SEM) was used to assess the associations between the total number of war events experienced, interpersonal sensitivity, and mental health outcomes (PTSD, depression/anxiety, and psychotic symptoms). Regression models were fitted in line with the strategies outlined by Baron and Kenny (1986) in SEM (Muthén & Muthén, 1998). A mixture of confirmatory factor analyses (CFAs) and multiple regressions were used to examine the relations between constructs in SEM. Constructs that are *unobserved* or *latent* variables (e.g., PTSD) are usually estimated by a factor analysis of data from theoretically related measures, such as *observed* or *indicator* variables (Muthén & Muthén, 1998). Accordingly, CFA was used to estimate latent variables for the mental health outcomes by loading the indicators from the PTSD, Depression/Anxiety, and Psychotic Symptoms scales. Each factor was identified by fixing the first item loading for each factor to 1, estimating the factor variance, and then fixing the factor mean to 0, while estimating all possible item thresholds (four for each item given five response options) and remaining item loadings. We used weighted least squares mean- and variance-corrected robust methods (all item residual variances were constrained to 1) and used a probit link and THETA parameterization to estimate all higher order models (Muthén & Muthén, 1998). Thus, model fit statistics describe the fit of the item factor model to the polychoric correlation matrix among the items.

The mediation models were assessed to examine the direct relationship between the number of war experiences and mental health outcomes and the indirect relationship between total number of war experiences and mental health outcomes via interpersonal sensitivity. All analyses were adjusted for sex. Models were fitted using the M*plus* software Version 7 (Muthén & Muthén, 1998). Mediation analyses is appropriate for this study because the WAYS study assessed previous war experiences retrospectively (more than 6 years ago), whereas interpersonal sensitivity and mental health outcomes were assessed at follow-up, slightly more than a year after baseline assessment.

## Results

Descriptive statistics and correlations of measures in the study are presented in Table 1. Generally, participants reported an average of 24.7 (*SD* = 6.1) traumatic war-related events, and 169 (37.3%) met diagnostic criteria for PTSD (≥33 on total PTSD score). The results of pairwise correlation analysis among study variables are presented in Table 1. All variables measured in the mediation model (war experiences, interpersonal sensitivity, PTSD, depression/anxiety, and psychotic symptoms) were significantly correlated with each other (Table 1).[Table-anchor tbl1]

### Confirmatory Factor Analyses

The CFAs indicated moderate-to-high loadings on their respective factors: Interpersonal Sensitivity (β = 0.60, *p* < .05 to β = 0.83, *p* < .0001), PTSD (β = 0.61, *p* < .05 to β = 0.85, *p* < .0001), Depression/Anxiety (β = 0.57, *p* < .05 to β = 0.87, *p* < .0001), and Psychotic Symptoms (β = 0.52, *p* < .001 to β = 0.89, *p* < .0001). The comparative fit indices ranged from 0.96 to 0.97 and root mean square error of approximation from 0.04 to 0.06. Comparative fit index values larger than 0.95 and root mean square error of approximation values below 0.06 have been suggested to indicate excellent model fit (Hu & Bentler, 1995; Kline, 2011).

### Measurement Model

**Figure 1 fig1:**
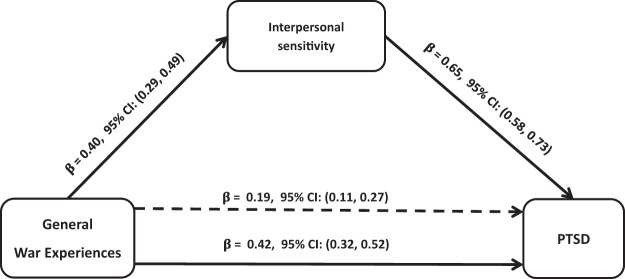
Mediation effects of interpersonal sensitivity on the relations between past war experiences and posttraumatic stress disorder (PTSD). Total effect: β = 0.42, 95% confidence interval (CI) [0.32, 0.52], and total indirect effect: β = 0.19, 95% CI [0.11, 0.27]. The β below the continuous line from war experiences to PTSD represents the total effect of war experiences on PTSD, whereas the β above the dotted line represents the effect of war experiences after interpersonal sensitivity was added to the model as a mediator. Approximately 55% of the effect of total number of war experiences on PTSD is mediated through interpersonal sensitivity. The direct effect of the total number of war experiences on PTSD was attenuated markedly but remained statistically significant, β = 0.19, 95% CI [0.11, 0.27]. All analyses were adjusted for sex.

**Figure 2 fig2:**
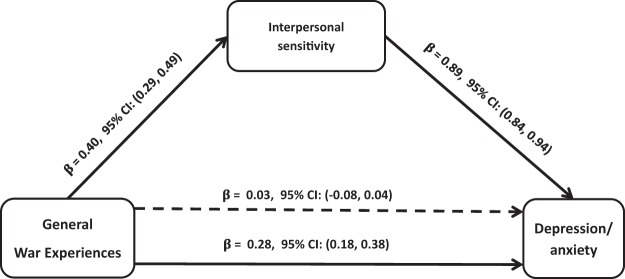
Mediation effects of interpersonal sensitivity on the relations between past war experiences and depression/anxiety. Total effect: β = 0.28, 95% confidence interval (CI) [0.18, 0.38], and total indirect effect: β = 0.03, 95% CI [−0.03, 0.04]. The β below the continuous line from war experiences to depression/anxiety represents the total effect of war experiences on depression/anxiety, whereas the β above the dotted line represents the effect of war experiences after interpersonal sensitivity was added to the model as a mediator. Apparently, the effect of total number of war experiences on depression/anxiety is fully mediated by interpersonal sensitivity. The direct effect of the total number of war experiences on depression/anxiety ceased to be significant, β = 0.03, 95% CI [−0.03, 0.04]. All analyses were adjusted for sex.

**Figure 3 fig3:**
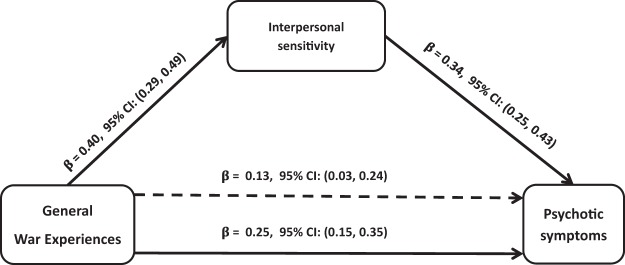
Mediation effects of interpersonal sensitivity on the relations between past war experiences and psychotic symptoms. Total effect: β = 0.25, 95% confidence interval (CI) [0.15, 0.35], and total indirect effect: β = 0.19, 95% CI [0.03, 0.24]. The β below the continuous line from war experiences to psychotic symptoms represents the total effect of war experiences on psychotic symptoms, whereas the β above the dotted line represents the effect of war experiences after interpersonal sensitivity was added to the model as a mediator. Approximately 53% of the effect of total number of war experiences on psychotic symptoms is mediated through interpersonal sensitivity. The direct effect of the total number of war experiences on psychotic symptoms attenuated markedly but remained statistically significant, β = 0.13, 95% CI [0.03, 0.24]. All analyses were adjusted for sex.

The SEM model, which assesses the strength of the direct relationships, indicated that there were significant direct associations between war experiences and PTSD (Figure 1), symptoms of depression/anxiety (Figure 2), and psychotic symptoms (Figure 3). Interpersonal sensitivity was statistically and significantly related to war experiences and to all indicators of mental health problems (symptoms of PTSD, depression/anxiety, and psychotic symptoms). Interpersonal sensitivity accounted for the relationship between war experiences and all indicators of mental ill-health by statistically significant indirect paths. For symptoms of PTSD, about 55% of the effect of war experiences is partially accounted for by interpersonal sensitivity. Interpersonal sensitivity fully accounted for the total effects of war experiences on symptoms of depression/anxiety. Similarly, 53% of the effect of war experiences on psychotic symptoms was partially mediated through interpersonal sensitivity. Whereas the effects of war experiences on symptoms of PTSD (Figure 1) and psychotic symptoms (Figure 3) markedly attenuated but remained statistically significant, the effects of war experiences on symptoms of depression/anxiety ceased to be significant after including interpersonal sensitivity in the mediation model (Figure 2).[Fig-anchor fig1][Fig-anchor fig2][Fig-anchor fig3]

Each regression coefficient represents the number of standard deviation (*SD*) change in the outcome variable per *SD* change of the independent variable. For example, the regression of interpersonal sensitivity on war experiences indicates that a change of 1 *SD* in the number of war experiences is associated with a 0.40 *SD* change in interpersonal sensitivity. When regression analysis was carried out between mental health outcomes and interpersonal sensitivity, after adjusting for war experiences, the proportion of explained variance increased from *R*^2^ = 0.19, *F*(4, 447) = 23.97, *p* < .001, to *R*^2^ = 0.47, *F*(4, 447) = 73.63, *p* < .001, for PTSD; from *R*^2^ = 0.08, *F*(4, 447) = 9.78, *p* < .001, to *R*^2^ = 0.78, *F*(4, 447) = 292.86, *p* < .001, for symptoms of depression/anxiety; and from *R*^2^ = 0.08, *F*(4, 447) = 9.06, *p* < .001, to *R*^2^ = 0.15, *F*(4, 447) = 14.91, *p* < .001, for psychotic symptoms.

## Discussion

The current study assessed interpersonal sensitivity as a mechanism that accounts for the relationships between war experiences and mental health problems—PTSD, symptoms of depression/anxiety, and psychotic symptoms—in formerly abducted youth in Northern Uganda. Interpersonal sensitivity fully accounted for the relationship between war experiences and depression/anxiety and partially accounted for the relationship with PTSD and psychotic symptoms. These results build upon previous findings with the same population, showing that postwar environmental stressors, stigma, and poor community relations are mechanisms by which war experiences impact mental health problems and functioning (Amone-P’Olak, Jones, Meiser-Stedman, et al., 2014; Amone-P’Olak et al., 2016; Amone-P’Olak et al., 2015; Amone-P’Olak et al., 2014). The results of this study corroborate previous findings suggesting that interpersonal sensitivity was related to PTSD symptoms (Allen, Coyne, & Huntoon, 1998; Huang, Zhang, Momartin, Cao, & Zhao, 2006) and various other mental health problems (Vidyanidhi & Sudhir, 2009; Wilhelm et al., 2004). Results of a longitudinal study with Vietnamese refugees who display PTSD symptoms showed markedly higher scores on interpersonal sensitivity than their peers who did not display PTSD symptoms (Hauff & Vaglum, 1994). It is worth noting in the current study that the indirect effects of war experiences through interpersonal sensitivity were stronger than the direct effect of war experiences on all the mental health outcomes. This indicates the important influence of the relationship between interpersonal sensitivity and mental health outcomes in the formerly abducted youth.

The influence of war experiences on PTSD and other psychopathology is well known, with previous studies indicating dose–response associations between war experiences and psychopathology (Mollica et al., 1998; Mollica, McInnes, Pool, & Tor, 1998). The findings of the current study demonstrate evidence for a possible mechanism with a potential to illuminate the path from war experiences to mental illness, suggesting that war experiences may lead to interpersonal sensitivity, which, in turn, may be linked to mental health problems. Considerable knowledge has so far been generated on the relationship between war experiences and mental health problems, but relatively little is known about the mechanisms underlying this relationship, especially factors related to postconflict environment (Amone-P’Olak et al., 2014; Fernando et al., 2010; Nickerson et al., 2014). The findings in this study provide modest evidence of the role of postwar environmental factors such as stressors, stigma, poor community relations, cognitive processes, and, indeed, interpersonal sensitivity. These findings may help to explain how war survivors become trapped in the path of long-term mental health problems and poor functioning.

Survivors of the war in Northern Uganda experienced horrendous war events: The majority witnessed violence, many were tortured, injured, involved in combat, used as human shields, and forced to mutilate, injure, or kill fellow abductees or civilians (Amone-P’Olak, 2004, 2009; Derluyn et al., 2004). This exposure to violence may limit trust and impact on daily functioning and social relations. Moreover, lack of trust is a common characteristic in postwar situations among survivors of violent conflicts (Solomon, Iancu, & Tyano, 1997).

One mechanism by which war experiences may be linked with postwar psychological distress may be through interpersonal sensitivity. Thus, previous studies have demonstrated that interpersonal sensitivity has an inverse correlation with one’s belief in positive human values and the goodness of fellow human beings, in the aftermath of disasters (Solomon et al., 1997). It is possible therefore that experiencing war atrocities and cruelty during war impairs trust and previous assumptions about the goodness of humanity (Nickerson, Bryant, Rosebrock, & Litz, 2014). This is consistent with the notion that adverse war experiences may challenge the previously held worldview that humans are inherently good (Solomon et al., 1997). Consequently, this leads to war survivors’ distrust of and hypervigilance to other people’s intentions to protect themselves and avoid further maltreatment (Foa, Zinbarg, & Rothbaum, 1992; Hagenaars et al., 2011; Nickerson et al., 2014). Furthermore, interpersonal sensitivity, mistrust, and other postwar cognitive processes are suggested to be long-term postwar outcomes (Beltran, Llewellyn, & Silove, 2008; Krysinska, 2010).

The findings that interpersonal sensitivity is a key determinant of the relationship between war experiences and mental ill-health have important implications. Previous research in Northern Uganda has indicated a high prevalence of anger and feelings of revenge among survivors in the aftermath of the war (Murphy et al., 2016, 2017). Moreover, anger, feelings of revenge, and aggression, in general, are likely to shape interpersonal and social interactions (Amone-P’Olak et al., 2016; Nickerson, Bryant, Rosebrock, & Litz, 2014). Theories of violence postulate that violence perpetuates further violence (Ryan, 2005). Consequently, interpersonal sensitivity may play an important role in explaining continued mental health problems and community violence in war-affected populations. Moreover, the LRA war in Northern Uganda was mainly fought against members of the community, often by people who spoke the same language as the victims and who may have been known to the victims as well. Moreover, the perpetrators were reintegrated into the same communities they once perpetrated violence against. As a result, there is a high level of distrust, personal vendetta, and sensitivity and a preoccupation with previous war experiences, which, in turn, engenders suspicion and constant perception of threats, leading to further mental health problems. Therefore, further studies are required to confirm the roles of interpersonal sensitivity in sustaining mental health problems. In addition, interventions to reduce mental health problems should consider interpersonal sensitivity as a strategy to reduce mental health problems.

The current study had numerous strengths. First, our sample was relatively large compared with previous similar studies (Amone-P’Olak, 2009; Amone-P’Olak et al., 2007; Derluyn et al., 2004; Kohrt et al., 2008). Second, we studied a large sample of difficult-to-reach war-affected youth who had been carefully enumerated by UNICEF, finding that 6 years after the end of the war, the lingering health problems are an indication of the toxic effects of the war on the mental health of the survivors. Finally, the SEM used in these analyses was robust and rigorous, thus reducing the effects of measurement errors in our constructs (Hu & Bentler, 1995).

Nevertheless, a number of limitations should be considered when interpreting the results of this study. First, it is possible that interpersonal sensitivity is a personality characteristic that existed before exposure to war events and is associated with psychopathology. Although the WAYS study is longitudinal, the existence of interpersonal sensitivity before the war cannot be ruled out. Second, war experiences were assessed retrospectively, thus rendering it prone to recall bias (Mollica, Caridad, & Massagli, 2007). Third, interpersonal sensitivity was assessed using a measure developed in the West; thus, whether it carries similar meaning may be limited by cultural differences. However, the internal consistency of the Interpersonal Sensitivity measure as used in the current study was acceptable at α = 0.82. Fourth, only four items were used to assess interpersonal sensitivity, which may be a limiting factor in robustly assessing this construct and its relationship to other variables in the study (Boyce & Parker, 1989). Finally, there could be other mediating and moderating variables not included in the current study that may explain the associations between war experiences and mental health outcomes in this study.

## Conclusion

The results of this study highlight the significance of assessing postwar contextual factors and adaptations in survivors of war. This study shows that interpersonal sensitivity is a key determinant of mental health problems reported by formerly abducted youth in Northern Uganda. It is worth noting that, at least for PTSD and psychotic symptoms, interpersonal sensitivity is not the only pathway contributing to differences in mental ill-health. Consequently, strategies to mitigate the noxious effects of war on mental health should consider factors beyond postwar contexts to include psychological care for formerly abducted youth and other war-affected populations who remain affected by the impacts of war (Yule, 2002). Postwar mental health should be predicated on a comprehensive approach that prioritizes postwar local contextual factors and psychological care to reduce mental illness among survivors. There are currently no particular interventions specifically designed for this population. Nevertheless, a number of local interventions based on indigenous knowledge and therapy (Amone-P’Olak, 2006) and clinical trials based on adaptations of Western therapy have been implemented with various degrees of success (Ertl, Pfeiffer, Schauer, Elbert, & Neuner, 2011; Sonderegger, Rombouts, Ocen, & McKeever, 2011).

Interventions such as cognitive–behavioral therapy to recognize and change cognitive schemas that make the youth vulnerable to interpersonal sensitivity are recommended. More specifically, culturally relevant cognitive–behavioral interventions that target the youth’s real or perceived negative appraisals of the behaviors or feelings of others and their personal response to such social situations should be developed. These interventions may include cognitive restructuring of negative schemas, relaxation, group interpersonal therapy, creative role-plays, and community altruistic activities (prosocial behaviors) such as helping disadvantages people (e.g., elderly and people with disability). Indeed, similar interventions to reduce psychological distress in the same population were able to reduce psychological distress (Amone-P’Olak, 2006; Ertl et al., 2011; Sonderegger et al., 2011). Group therapy would be culturally appropriate for the youth for two reasons: first, because it is conducted in a group setting, it would create social support, and second, it would reduce stigma associated with individualized mental health interventions, which many of the youth would shy away from for fear of being labeled by peers and other members of the community. Finally, more research is needed to unravel the mechanisms by which war experiences account for mental illness, especially in low-resource settings with a view of developing strategies to improve mental health of war-affected populations.

## Figures and Tables

**Table 1 tbl1:** Descriptive Statistics and Correlations of Measures in the Study

Descriptive statistics	*M*	*SD*	Minimum–Maximum	1	2	3	4	5	6	7	8	9	10
1. Age at baseline	22.39	±10.47	18–25	—									
2. Duration in captivity	3.48	±3.40	.5–15	.12*	—								
3. Interpersonal sensitivity	2.79	±2.99	00–16	.13*	.18**	—							
4. Arousal	17.91	±2.73	00–28	.15**	.15**	.72**	—						
5. Avoidance	19.14	±3.13	00–32	.13*	.11*	.46**	.62**	—					
6. Intrusion	17.88	±2.19	00–28	.10*	.19**	.56**	.65**	.56**	—				
7. Total PTSD symptoms	54.61	±6.94	00–88	.15**	.17**	.67**	.88**	.87**	.83**	—			
8. Depression/anxiety	21.29	±10.47	00–54	.09	.14**	.88**	.33**	.25**	.35**	.35**	—		
9. Psychotic symptoms	5.66	±1.43	00–16	.11*	.15**	.37**	.30**	.18**	.25**	.28**	.32**	—	
10. Total war experiences	41.71	±4.19	00–52	.31**	.25**	.40**	.40**	.35**	.37**	.43**	.28**	.27**	—
*Note.* PTSD = posttraumatic stress disorder. Significant correlations are indicated in bold.													
* *p* < .01. ** *p* < .001.
